# The shift from quantitative to qualitative thinking—problems and prospects as viewed from Husserl’s and Hegel’s philosophy

**DOI:** 10.3389/fpsyg.2023.1232420

**Published:** 2023-09-18

**Authors:** Christopher Gutland

**Affiliations:** Department for Philosophy, Zhejiang University, Hangzhou, China

**Keywords:** qualitative thinking, experience of thinking, methodology, quantification, measurement, consciousness, Husserl, Hegel

## Abstract

This article contrasts the views of the philosophers Husserl and Hegel on quantification in science and compares their proposals for conducting rigorous qualitative research. Both deem quantification integral to science, but furthermore proposed methodologies to investigate qualitative necessities achieved by a shift in conscious activity and awareness. However, their methodologies differ significantly. While Husserl rejects idealization and instead proposes intuitive means to ideate qualitative essential relations, Hegel suggests idealizing less one-sidedly, namely, qualitatively over and above quantitatively. The article first examines how quantification is achieved and how it contrasts with measuring. This contrast reveals that measuring implies knowledge of qualities. These qualities, however, thus far remain oddly external to the mathematical relations linking the various established equations. The article then follows Husserl’s reconstruction of the development of science to illustrate the dismissal of many experiential qualities and how philosophy further amplified skepticism about science on qualities. Husserl’s notion of the life-world and the method of eidetic variation are then introduced as means to counterbalance mathematical proceedings in science. However, this method reveals both eidetic qualitative structures and psychical structures without being able to distinguish between them. It is thus susceptible to idiosyncratic, traditional, and cultural biases. Subsequently, Hegel’s description of the shift in conscious experience that sets qualitative from quantitative thinking apart is introduced. This shift may overcome the biases, but it faces skepticism that calls for further investigation of the experience of different kinds of thinking.

## 1. Introduction

This article contrasts the viewpoints of the philosophers Edmund Husserl and Hegel on how quantification as a methodological yardstick of scientific objectivity relates to qualitative research. Both deem quantification integral to science but suggest methodological means to further explore the essential qualitative aspects of reality. Curiously, both propose that the study of these qualities involves a certain shift in conscious activity and awareness. Nonetheless, their respective methodological proposals are very different. After discussing the techniques of quantification and measurement, this article compares these proposals.

Consistently portraying the two philosophers’ views requires two terminological clarifications. First, what Hegel and Husserl call “science” (*Wissenschaft*) encompasses natural sciences, social sciences, arts, and the humanities. Since the historical split between natural science and the humanities is a topic here, “science” here is similarly used as an umbrella term. Whenever only natural science is meant, it is referred to as “natural science.” Second, this article follows Hegel (1986c, p. 44) in understanding “mathematics” strictly as the science of quantity. This needs to be emphasized because some subsume qualitative concepts when they speak of “mathematics.”^1^

Given the hostility toward mathematics occasionally encountered in the phenomenological tradition, it is worth highlighting: This article’s goal is not to abandon measuring and quantification. That would only substitute one one-sidedness for another. Quantification brought unquestionable merits. The question, instead, is how the focus on quantity may be complemented by methodologically integrating quality. It is also worth noting that the focus is not on qualitative research in general but specifically on *conceptual* or *essential* qualitative relations.

The second section examines the process of quantification and how measuring transcends quantification because it depends on qualities. This dependence and integration of qualities in scientific measuring are then investigated further. With Husserl, the historic scientific dismissal of consciously experienced qualities is traced along with his suggestion of where to find and how to investigate reality as still consisting of these qualities. This will reveal shortcomings in Husserl’s suggestion. Lastly, how Hegel proposes to integrate the qualitative understanding of nature and consciousness methodologically will be considered.

Given the format of an article, the focus lies on unveiling and contrasting the views as expressed in the primary texts while drawing occasional links to scientific proceedings today. The outlook section then sketches how qualitative thinking relates to more recent scientific approaches.

## 2. Background: quantification and measurement

### 2.1. Pure quantification

When critically revising a habit, Spinoza (2018, p. 231) suggests to first “focus on what is good in” the habit as it is. In this sense, a striking merit of mathematics and quantitative thinking is their remarkable immunity to the influences of cultural, national, or historical differences and biases. Unlike in philosophy and religion, we do not distinguish between Indian, Chinese, and European mathematics. Instead, mathematics is a common ground wherein people from all cultures can equally participate, contribute, and cooperate. No one is excluded because of their culture, nation, language, or history, and neither can anyone point to these factors to claim themselves superior to others.

Given its goal of achieving objective and valid truths while avoiding biases and prejudices, it is easy to see why mathematics became a scientific yardstick. Kant (2004, p. 6) famously held that “in any special doctrine of nature there can be only as much *proper* science as there is *mathematics* therein.” It is characteristic to this day to consider as scientific only what can be quantified. Only few take serious suggestions like Wittgenstein’s (1974, p. 414) that mathematics might be relative to language and result from conditioning by one’s teacher. Yet why is mathematics so immune to cultural and historical biases? And why are such factors so prone to meddle with philosophical and religious reasoning?

The answer lies in the degree of abstraction required when quantifying something. Husserl (1970a, p. 145, 2003, p. 153)^2^ gives a striking example: “To the question, ‘How many are Jupiter, a contradiction, and an angel?’ we immediately answer: ‘Three.’ […T]he units are ‘the same’ as each other. But these samenesses of theirs are a *consequence* of number abstraction, not its basis and presupposition. They arise, not through a preliminary comparison, but rather through that absolute depletion of content which number abstraction requires under all circumstances.” That is to say: *To count something, we must abstract from all its qualities*. The only remaining property is that whatever was counted is a “one.” In this way depleted of its qualitative content, we can then combine it with *any other* quantifiable content, no matter how qualitatively unrelated they are.

After all, the qualitative differences between an angel, Jupiter, and a contradiction might make it difficult to see any reason to group them together. Mathematically, however, we may relate them by adding them up, and then they *are* three. This abstraction from qualitative content is thus a reason why mathematics overcomes cultural differences. Different cultures have very different notions of an angel. Yet no matter whether someone assumes angels to exist or not, whether they deem them real, ideal, metaphysical, good, or evil—everyone seems to agree that if counted, an angel is “one.” As such a “one” it can be added at will to further instances of “one,” yielding “two,” “three,” etc. Whatever else an angel is beyond a “one” is ignored, and the mere oneness instills no cultural disputes.

Hegel (1986d, p. 244, 2010, p. 178) similarly explains that a number’s “element is the difference which has become indifferent.” Like Husserl, Hegel (1986d, p. 80, 2010, p. 56) emphasizes that the “one” is not the basis of thinking, but rather an advanced abstractive result: It “is clear from a comparison of quality with quantity that the former is by nature first. For quantity is quality which has already become negative; *magnitude* is the determinateness which […] is the sublated quality that has become indifferent.” Hegel (1986d, p. 91, 2010, p. 65) adds, “*numbers* are neither the first simple, nor the self-abiding thought, but thought rather which is entirely self-external”. The self-externality of mathematical thinking is picked up further down again.

Mathematics in and of itself can explore the relations of quantities in dependence on a select number of presupposed axioms. Yet the endeavor of science is more expansive than such pure mathematical exploration. Science *applies* mathematics to the world. By applying it, however, it already transcends quantity and inevitably re-enters the sphere of qualities.

### 2.2. Exceeding pure quantification via measurement

We apply mathematics to our world whenever we measure. However, measuring is not a purely quantitative process. It is only possible drawing on qualities. In measurement, quantities are related to qualities like length, volume, speed, or mass. Measuring is geared toward such qualities and determines the quantity of a related unit within the measured quality. For instance, we measure a certain length (quality) by determining how many (quantity) inches (unit) it contains. The unit is thus a necessary fulcrum for measuring: It defines a certain quantity of quality to count as “one.” For many units, there is quite some latitude to standardize this “one.” An illustration for this latitude is the contrast between the imperial system (using inches, feet, miles, etc.) and the metric system (using centimeters, meters, kilometers, etc.).

Thus, if we take Kant’s view literally, proper science must not measure, because the quality inevitably entailed in measuring is beyond mathematics. As an illustration, take Einstein’s famous equation $E=m\;c^{2}$. Mathematics is used here merely as a means to *express* a particular relation between the variables E, m, and c. These variables represent qualities measured in specific units. In Husserl’s (1962, p. 40, 1970b, p. 41) words mathematics is used to “express general causal interrelations, ‘laws of nature,’ laws of real dependencies in the form of the ‘functional’ dependencies of numbers.” Because of the abstraction from all content that is more specific than “one,” however, these variables all represent pure quantities. Consequently, the related qualities as such cannot enter mathematics.

Husserl (1962, p. 44, 1970b, p. 44) describes a consequence of this when we are doing geometry purely arithmetically: “In algebraic calculation, one lets the geometric signification recede into the background as a matter of course, indeed drops it altogether; one calculates, remembering only at the end that the numbers signify magnitudes.” This observation is valid for any calculation containing variables representing qualities. *During the calculating* of an equation like Einstein’s, we *must* leave out what qualities (and their related units) the quantities stand for. Because of this, once we succeed in calculating the result, we may have to look up what quality the resulting figure stands for. Quality cannot enter this kind of thinking.

Scientists are quite aware of the related temptation to only mind the quantitative relations. An introductory physics book seeks to impress on us: “The measurement of any quantity is made relative to a particular standard or *unit*, and this unit must be specified along with the numerical value of the quantity. […] To specify that the length of a particular object is 18.6 is meaningless. The unit *must* be given” (Giancoli, 2014, p. 12). However, mathematically speaking, units like μL or km are meaningless. *Only* a figure like 18.6 is meaningful. The strong accentuation that “the unit *must* be given” is thus not least to compensate for the need to abstract from all quality during the calculating.

Consequently, mathematics cannot know or process what energy or mass qualitatively *are* in an equation like $E=m\;c^{2}$. They can be processed only insofar as they are quantifiable. However, on this pure mathematical level, any “one” can be added to any other “one” to form two “one.” The need to be mindful of the unit also relates to this. For in physics, we must pay attention not to sum up a “one” that represents a length’s unit with a “one” representing an impulse’s unit.

To this day, the mathematical structures encountered in science remain in the way described *detached* from the measured qualities. Even though the qualities are usually related to some variable’s letter (like “m” for mass or “v” for velocity), whenever mathematically processing them, the respective numbers get detached from these qualities. If we could *think* such that *our thinking included* these qualities, we would neither forget them during the thinking nor need to be reminded to indicate their unit. The later sections of this article ponder the possibility for such a thinking.

From what was observed also follows that there is no mathematical reason why $E=m\;c^{2}$ is true. If our world were such that instead E=(1/4) m−c or $E=\sqrt[{m}]{c}$ were correct, mathematics would be just as apt to express it. Thus, the specific way the variables relate to each other, albeit mathematically *expressible*, is not the way it is for mathematical reasons. If we want to know which of the three equations is true, we again need to go beyond mathematics.

What is possible, of course, is to mathematically transform equations that have been established. For instance, if $E=m\;c^{2}$ is correct, then $E/m=c^{2}$ is also correct. *This* correctness is mathematical. For these transformations are correct *irrespective of the qualities* E, m, and c. They would be correct even if $E=m\;c^{2}$ would not be true.^3^

One can furthermore mathematically relate $E=m\;c^{2}$ with other equations containing E, m, or c, thereby “discovering” otherwise not yet established equations. That such transformations tend to conform with empirical reality can be considered remarkable. It shows that the mathematical relations we experience purely in our minds are somehow woven into the constitution and fabric of external reality. Nevertheless, we first need to non-mathematically establish a set of such equations. As was shown, we cannot establish them purely based on mathematics.

The point of this section was to show that although qualitative elements are quantitatively related within equations, their qualitative meanings remain external to this processing. In Hegel’s (2010, p. 234) words: “[M]athematics is in principle incapable of demonstrating the quantitative determinations of physics, for these determinations are laws based on the *qualitative nature* of its elements.” Husserl (1962, p. 40, 1970b, p. 41) likewise remarks about equations that “their true meaning does not lie in the pure interrelations between numbers (as if they were formulae in the purely arithmetical sense).” Hegel (2010, p. 234) adds “as long as it is not clear about the distinction between what can be proved mathematically and what can only be taken from elsewhere, […] scientific culture will lack rigor and purity.” His view on how qualitative relations can be investigated with the same rigor as mathematical ones will be developed further down.

Yet if measuring is impossible without relying on qualities, but knowledge of qualities cannot stem from mathematics, then how does science acquire knowledge about qualities?

## 3. Science and empirical observation

In the last quote, Hegel said that knowledge about qualities has to be ‘taken from elsewhere.’ What is this elsewhere?—Hegel (2010, p. 298) writes: “Proofs of this kind *presuppose* their theorems and even the laws to be proved from experience; what they manage to accomplish amounts to this, that they reduce such theorems and such laws to abstract expressions and convenient formulas.” The non-mathematical source of knowledge about qualities is thus experience, namely in the form of empirical observation. Based on empirical observation, science establishes, confirms, and reassess its formulas and theories. This principal dependency on experience remains unaltered even if it is used to falsify (Popper, 2002) rather than verify theories.

However, since Hegel’s days, science abandoned Bacon’s inductivism and switched to hypothetical-deductive theory building (Carrier, 2009, pp. 17–19). That means: It is now allowed to assume unobserved hypothetical elements in theories. A theory that postulates such elements is accepted if the observable events that follow deductively from the acceptance of the theory are consistent with actual empirical observation. Such lenient criteria^4^ for theory building lead to Duhem-Quine underdetermination: Two or more theories assuming different unobserved elements may equally well predict observable events. An example from physics is Bohm’s mechanics versus the standard model of quantum mechanics (Carrier, 2009, p. 20).

However, this article does not delve into the issues due to allowing entirely hypothetical elements in theories. It needs to be mentioned because it entails that scientific theories not only take up qualitative elements from actual observation but may also contain hypothetical elements. Nevertheless, even in hypothetical-deductive reasoning, experience or empirical observation has “the last word” insofar as observations contradicting a theory’s predictions falsify the theory.

But how did science’s use of experience and the establishment of mathematics as its yardstick develop historically?

### 3.1. Husserl on the idealization implied in measuring with geometric idealities

In his reconstruction of modern science, Husserl focuses on Galilei and sees his principal achievement as a “*mathematization of nature*” (Husserl, 1962, p. 20, 1970b, p. 23). Husserl observes that before Galilei, people knew about the individual differences in experiencing the world, yet nonetheless naively assumed all experience the same world (Husserl, 1962, p. 20, 1970b, p. 23). Measurement for the first time substantiated this assumption as it allowed to determine certain properties such that the outcome was *the same* for *all*, i.e., objective (Husserl, 1962, p. 25, 1970b, pp. 27–28).

Yet the initial measurements had two prerequisites. The first are standardized units like meters or inches. Secondly, they require knowledge of geometric idealities like straight lines or squares. Say we wish to measure a length. Then we need both an established *unit* like meters and a way to conceive of the length’s *form*. After all, a mere length does not specify its form, and neither does its unit. When we measure a broom’s length, we use the ideal geometric shape of a straight line. If we measure a tire’s circumference, we do so by conceiving of it as a circle.

Husserl puts much critical focus on the required geometrical idealities, interpreting them as an idealization that estranges us from the experienced reality. For instance, Husserl (1962, p. 21, 1970b, p. 24) requires to “separate the space and the spatial shapes geometry talks about from the space and spatial shapes of experiential actuality.” He stresses that even in imagination, we cannot intuit geometric ideality (Husserl, 1962, p. 22, 1970b, p. 25). Consequently, for Husserl (1962, p. 22, 1970b, p. 25), “geometrical space does not mean anything like imaginable space.” This distinction between ideal and intuitive space at the root of Husserl’s critical attitude will be picked up again further below.

Husserl thus believes scientific theories are *not directly about* the world we experience: “In geometrical and natural-scientific mathematization […] we measure the life-world—the world constantly given to us as actual in our concrete world-life—for a well-fitting *garb of ideas*, that of the so-called objectively scientific truths” (Husserl, 1962, p. 51, 1970b, p. 51). If we measure the area of a cornfield, we objectively obtain a mathematically exact figure. However, Husserl assumes the experienced world to be inexact. Therefore, he believes that through measuring—due to the employed idealities—we inevitably exit the inexact experienced world and enter an idealized one. This is why Husserl believes that when we measure, rather than getting to know the world we experience more intimately, we get estranged from it.

That measuring with lines, areas, and volumes depends on geometrical idealities is frequently overlooked. Even less noticed is that these ideal shapes are still *qualitative*. That means: Strictly speaking, geometry supersedes mathematics as defined by Hegel. Husserl critically notices the same point when describing the ‘arithmetization of geometry.’ Therein, the “spatiotemporal idealities […] in geometrical thinking” become “pure numerical configurations,” thus undergoing the same “emptying of meaning” discussed above (Husserl, 1962, p. 44, 1970b, p. 44).

Before discussing the problems of measuring qualities, it is useful to briefly illustrate how mathematical functions are used in science. This allows to better contrast the different acceptance criteria for quantitative versus qualitative idealities.

### 3.2. The tolerance toward geometric idealities and mathematical functions

**Figure 1:**
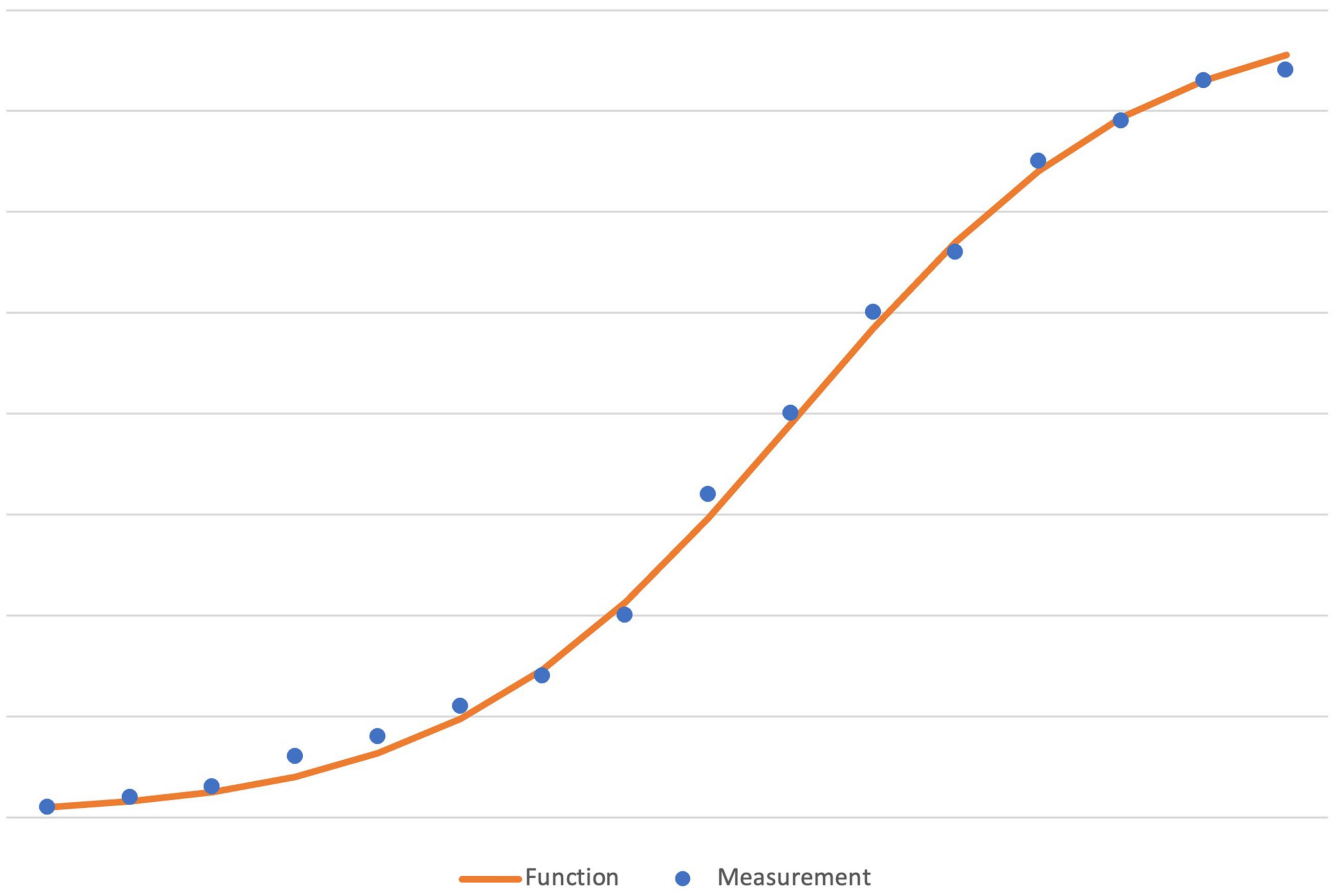
An illustration of the typical deviations between actual measurements and an ideal curve (here: the logistic function).

A suitable example is to look at how biology estimates the growth of a bacteria population in an environment of limited resources. The logistic function is used to model such growth. As a result, we may encounter a graph like this:

In this diagram (Figure 1), the blue dots represent the measurements and the orange line the ideal logistic growth curve. Notice how almost all dots are slightly above or below the mathematical curve.^5^ In spite of this deviation, a scientist still uses the curve to model the actual growth within the scientific calculations. It is, after all, well known that—over and above the inevitable measurement errors—there are always factors that cannot all be controlled.^6^ Therefore, some deviation of the actual measurements is accepted and may be indicated in the graph as the standard deviation. We may read sentences like: “The logistic model fits few real populations perfectly, but it is useful for estimating possible growth” (Urry et al., 2021, p. 1212).

Thus, even if no measurements are exactly on the curve, they can be seen as *confirming* the appropriateness of the curve, rather than a reason for dismissing it as a mere fiction. Put another way: The *individual* measurements, despite their distance from the curve, are accepted as confirming the *ideal* curve. When pressed hard about the deviation, a scientist may say these lines and curves are “only models.” Nonetheless, such models are powerful tools of prediction. Let us now first turn to what could not be measured with geometrical idealities.

### 3.3. The dismissal of our subjective conscious experience from objectivity

For quite some time, the idea that the entire world is but a complex mathematical system had to wait. The reason was that it is not possible to measure *everything* we experience with geometrical shapes. Until Galileo, aspects of our conscious experience, namely colors, tones, smells, tastes, temperatures, and odors, escaped scientific measurability. Locke (1997, p. 135) called them the “secondary qualities.” Because they, so to speak, “fill out” the pure geometric shapes, Husserl calls those qualities “plena” (*Füllen*). Although these qualities are experienced in graduality—as more or less sweet, bright, sharp—measurements were at first impossible (Husserl, 1962, p. 32, 1970b, p. 34). What was missing were “particular ideal structures,” akin to the geometrical ones when measuring lengths, “that can be correlated with given scales of measurement” (Husserl, 1962, p. 33, 1970b, p. 34). For instance, when we look at a table, we easily think of various geometric forms that would allow to measure its dimensions in space. Nevertheless, we seem to lack similar ideal forms that would allow to measure the table’s color. Thus, the project to objectively determine all our experience through measurement stalled whenever confronted with these qualities.

However, these qualities *indirectly* correlate with *other* phenomena that are measurable with geometric shapes. Already “the ancient Pythagoreans had been stimulated by observing the functional dependency of the pitch of a tone on the length of a string set vibrating” (Husserl, 1962, p. 36, 1970b, p. 37). The quality of such a tone escapes measurement with geometric idealities, yet the swinging string’s length, amplitude, and frequency do not, and the tone quality is reliably related to them. Such an *indirect* access to measure more and more plena was discovered. This paved the way for the bold hypothesis of a “universal idealized causality” that “encompasses all factual shapes and plena in their idealized infinity” (Husserl, 1962, p. 38, 1970b, p. 39).

Soon, however, whatever was only indirectly measurable was dismissed as “merely subjective” in the sense of non-objective. Husserl (1962, p. 54, 1970b, p. 54) mentions “Galileo’s famous doctrine of the merely subjective character of the specific sense-qualities, which soon afterward was consistently formulated by Hobbes as the doctrine of the subjectivity of all concrete phenomena of sensibly intuitive nature and world in general. The phenomena are only in the subjects; they are there only as causal results of events taking place in true nature, which events exist only with mathematical properties. If the intuited world of our life is merely subjective, then all the truths of pre- and extra-scientific life which have to do with its factual being are deprived of value. They have meaning only insofar as they, while themselves false, vaguely indicate an in-itself which lies behind this world of possible experience and is transcendent in respect to it.” That is to say our consciousness presents the object to have qualities like colors and tones that are not objective properties. Dismissing the specific sense qualities from objective reality like this means: *Our conscious experience of the world is not only flawed; it betrays us*.

What does it mean that these “objectively inexistent” qualities are “in the subjects.”—Galilei (1957, p. 274) elaborates: “I think that tastes, odors, colors, and so on are no more than mere names so far as the object in which we locate them are concerned, and that they reside in consciousness.” Newton (1952, pp. 124–25) similarly stressed about light rays: “Rays, to speak properly, are not colored. In them there is nothing else than a certain Power and Disposition to stir up a Sensation of this or that Color.” Views like these render our conscious experience only partly trustworthy, but on many counts as misleading or outright false.

It is worth noting that science here had it both ways: It considered the merely *indirect integration* of the qualia as reason enough to *accept* its thoroughly mathematical worldview. However, at the same time it *excluded* these qualities from objective nature as if their integration had failed. One of the many consequences of this inconsistent inclusion–exclusion is the debate on qualia (Tye, 2021). For instance, in the Mary’s Room thought experiment, the question is whether a color qualia is reducible to the objective (neurophysiological) processes involved (Jackson, 1986). Due to the dismissal of secondary qualities as non-objective, the very awareness that there might be a method to directly integrate them faded. Science began to contrast objectivity with conscious subjective experience.

Up to this point, science’s relation to qualities is thus threefold: It (1) takes up knowledge about certain qualities through empirical experiments, (2) adds hypothetical elements to its theories, and (3) excludes only indirectly measurable qualities from objective reality.

To be fair: in his portrayal, Husserl leaves out other reasons that seemingly jeopardize the specific sense qualities’ objectivity.^7^ However, today still, science frequently tries to go beyond (transcend) our “subjective” consciousness to find the “real” properties and processes. Husserl (1960, p. 24, 1973a, p. 63) sees Descartes as the father of this “transcendental realism.” A look at Descartes and Hume indeed reveals the considerable philosophical support for the dismissal of qualities.

### 3.4. Philosophical attitudes toward qualitative idealities before Husserl and Hegel

Descartes strived to replace Aristotelian physics with a mechanistic approach (Moriarty, 2008, p. xix). For Aristotle, the sensory quality of heat corresponds to an objective property, while Descartes (2008, pp. 58, 240) sought to reduce it to bodily movements instead. Descartes casts doubt on the sensory qualities as a reliable basis for objective knowledge. He sees them as merely helpful to preserve our bodies (Descartes, 2008, pp. 51–64). However, Descartes (2008, p. 51) maintains that “the whole of this bodily nature which is the object of pure mathematics […] can be plainly known to me with certainty.” With a view like this, he inspired not only transcendental realism but also views like that of Kant’s on mathematics.

Hume (2007b, pp. 45–46) famously required that thoughts or ideas be made clear based on experience. However, Hume (2007b, p. 18) notably excluded “Geometry, Algebra, and Arithmetic,” claiming their propositions are “discoverable by the mere operation of thought, without dependence on what is any where existent in the universe.”

Let us—for a moment—assume Hume had also required geometry, algebra, and arithmetic to strictly base their ideas on experience. Then, a biologist could only use the logistic function to predict population growth if *all* previous measurements were *exactly* on the function. Otherwise, our experience would have been no basis to even think the idea of the logistic function. Consequently, such mathematical functions would be unusable. Experience would need to be interpreted such that it, if anything, proves the inexistence and thus inadequacy of this function. Where we even obtained the idea of the function would be an enigma. Hence, if Hume’s requirement for a sensory basis for idealities were made a criterion for all science, we could not use mathematic idealities in science.

This shows how Hume’s philosophy helped establish a double standard regarding quantitative and qualitative idealities. After Hume, qualitative idealities had to pass a test that mathematical idealities would fail. However, Hume did not prove at all that qualitative idealities are not just as well ‘discoverable by the mere operations of thought.’ The fifth section will pick up a suggestion that they can.

For finding out which features essentially belong to something, Hume suggested to proceed inductively. And Hume (2007a, p. 62) is quite right claiming “there can be no *demonstrative arguments* to prove, *that those instances, of which we have had no experience, resemble those, of which we have had experience*.” To use the standard example: Even if all swans we saw were white, this would be no ground to assume all individual swans are white. However, this is trivial on the level of individuals. Hume’s assumption gets problematic if it is interpreted such that knowing the *totality* of *individual* swans would reveal *essential* features of a swan. There are at least three reasons why the quantity of observed individuals, even if complete, does not warrant knowledge of essential or ideal properties.

The first is that the presence of a certain quality in *all individuals* (i.e., a particular quantity) is no sufficient condition to know whether this quality is *essential*. Put another way: essential quality is irreducible to the quantity of individual totality. The second section of this article demonstrated the impossibility to know quality based on quality, since in order to know quality, we need to abstract from all quality.^8^ Hegel (1986a, p. 111/§39, 1991, p. 80) therefore criticizes Hume: “It is true that empirical observation does show many perceptions of the same kind, even more than we can count; but universality is altogether something other than a great number.”

Second, Hegel (1986b, pp. 34–36/§250) points out that *deviations* of an individual’s features from those *essentially* belonging to it—like the measurements usually slightly deviating from the logistic function—*occur for all essences (universals)*. Therefore, he suggests abandoning the requirement of a *complete alignment* of essential features with empirical ones not only for mathematical functions, but also for qualitative essential features. How Hegel instead believes we can know essentiality is discussed further down.

The third problem is that the supposed result of induction is instead presupposed at its outset. The problem can be summarized in the question: *If all swans we saw were white, and then we see a black bird, how do we know if it is a swan*? Hume’s induction naively presupposes that *if* the black bird we see *is* a swan, *then we will know*. But on what basis do I make this judgment? Answering *this* question, i.e., *how I know a black bird I have never seen is a swan even though all swans I saw before where white* seems much more promising than the tedious observation and protocolling of all swans and their features. For this categorizing ability is naively presupposed to perform this kind of protocolling.

This is precisely where the qualities underlying our everyday life are overlooked. We may furthermore note that a black swan and a one-winged swan relate to our underlying notion “swan” very differently. We may feel pity for the swan with one wing, realizing it is deprived of something essentially belonging to it. However, we feel no similar pity that the black swan is not white.

At this point it may seem as if the qualitative idealities that help us seeing a swan as a swan are all already present in our everyday life, unnoticedly underlying it. If so, the problem would merely be to find a means to bring them to light. Yet is this so? At times, Husserl seems to advocate this with his notion of the life-world.

## 4. Husserl’s life-world ripe with qualities

To better understand Husserl’s critique of science, it is worthwhile to point out an underlying chicken or the egg problem. Science usually explains consciousness as based on objective processes: First, we need physical matter. Afterward, there can be life, then consciousness. Consciousness is thus conceived of as only possible based on physical matter (sometimes even as entirely reducible to it). Husserl’s view is quite the opposite. Not, however, because Husserl thinks there could be no matter without consciousness. Instead, he wishes to be mindful of our epistemological starting point. He believes that however much science abstracts from or belittles conscious experience, science *as a human practice* is inevitably *situated in it*. Husserl (1962, pp. 48–54, 1970b, pp. 48–53) assumes that scientific measuring, abstracting, and theorizing is something we constantly—albeit unnoticedly—*perform within consciousness*. For Husserl, consciousness thereby becomes a necessary condition for *doing* science. He views science is an evolving construct, a web of meanings developed in and based on consciousness. That is why for Husserl, abandoning conscious experience as unreliable reveals a methodological lack of self-awareness. For it is *in and based on* consciousness that we first learn about science, understand it, then rethink and develop it further.

Nonetheless, Husserl views it as only natural that our consciousness’s role in getting to know the world was almost constantly overlooked. He even calls the attitude of being only interested in worldly things and their existence the “natural attitude.” He contrasts it with the *epoché* as a shift in conscious awareness that allows us to instead note how we are *conscious of* the world (Husserl, 1976, pp. 56–134). Husserl (1962, p. 204, 1970b, p. 200) calls this shift of consciousness required to investigate consciousness a “complete inversion of the natural stance of life, thus into an ‘unnatural’ one.” Within this shift of conscious awareness, we can examine how our normal conscious awareness functions as the foundation of scientific theory building.

Husserl calls the “world” that existed before science and that implicitly underlies all scientific practice the “life-world.” Many phenomenologists emphasize the founding role that the life-world played according to Husserl (Staiti, 2017, p. 177; Ströker, 1979; Sowa, 2010). For instance, when we enter a laboratory, we do not leave the life-world behind and experience scientific objectivity. When we experience the instruments, probes and colleagues and interact with them, we continue experiencing qualities like colors, sounds, smells, and even use them to orient ourselves. We do so when we look at a chromatography’s color distribution, listen to the sounds of a Geiger counter, or detect by a pungent smell that oxygen transformed into ozone. Husserl (1962, p. 128, 1970b, p. 125) stresses that “to use the life-world in this way is not to know it scientifically in its own manner of being.” An unbiased investigation of how natural science’s theoretical attitude to experiential qualities relates to its practical reliance on them is still a desideratum.

Yet Husserl not only views the life-world as science’s foundation, but also as its “ultimate purpose which the new science […] growing out of prescientific life and its surrounding world, was from the beginning supposed to serve: a purpose which necessarily lay *in* this prescientific life and was related to its life-world. Man (including the natural scientist), living in this world, could put all his practical and theoretical questions only to *it*—could refer in his theories only to it” (Husserl, 1962, p. 50, 1970b, p. 50). Even today, we constantly raise questions based on how we consciously experience the world and relate science’s answers to it as well.

For example, even those who believe colors are not objective often still care about the color of their car or cell phone. Most have found ways to seamlessly switch between the opposition of our conscious experience of the world and what science claims it “really” comes down to. Others even suggested we can learn to ignore experiential reality. For instance, Brown (1992, p. 357) writes: “[O]nce we grasp the correct scientific account of an item, we can respond to stimuli directly in terms of the concepts of that account. Instead of describing a physical object as red, we can learn to describe it as reflecting light of wavelength 6,300 angstroms—and we can do so without conscious inference […]. Thus, in the long run, we will be able to describe physical objects immediately and directly in terms of their intrinsic properties.” To my knowledge, however, such efforts have not borne fruit.

Husserl (1962, p. 52 1970b, pp. 51–52) explains his own view of this relation as follows: “Mathematics and mathematical science, as a garb of ideas, or the garb of symbols of the symbolic mathematical theories, encompasses everything which, for scientists and the educated generally, *represents* the life-world, *dresses it up* as ‘objectively actual and true’ nature. […]. It is because of the disguise of ideas that the true meaning of the method, the formulae, the ‘theories,’ remained unintelligible and, in the naive formation of the method, was *never* understood.” Thus, while science renders our conscious experience a deceitful disguise of the objective processes underlying it, Husserl instead counters that science deceives us by making us believe the world objectively is something we do not experience.

Yet what it is that, for Husserl, sets apart the ideal entities that his phenomenology explores from those that science explores? He writes: “Plainly the essential forms of all intuitive data are not in principle to be brought under ‘exact’ or ‘ideal’ notions, such as we have in mathematics. The spatial shape of the perceived tree as such, taken precisely as a ‘moment’ found in the relevant percept’s intentional object, is no geometric shape, no ideal or exact shape in the sense of exact geometry. […] The essences which direct ideation elicits from intuitive data are ‘inexact essences,’ they may not be confused with the ‘exact’ essences […] like an ‘ideal point’, an ideal surface or solid [… which] arise through a peculiar ‘idealization.’ The descriptive concepts of all pure description, i.e., of description adapted to intuition immediately and with truth and so of all phenomenological description, differ in principle from those which dominate objective science” (Husserl, 1984a, p. 249, 2001b, p. 15).

Husserl here again distinguishes the two spaces mentioned above. He suggests that phenomenology, within its *ideation* of the essential structures underlying conscious experience, stays faithful to this experience. The price it pays for this faithfulness is “inexactness.” On the other hand, science achieves exactness but pays the price of unfaithfulness to the experience that underlies its *idealizations*. Another way of putting this would be to say that our life-worldly experience does not contain the ideal entities that result from idealizing. Instead, it contains other ideal entities—essences or eide—which can be investigated and clarified through ideation.

This supposed distinction of ideation and idealization, among other things separating intuitive and ideal space, however, is not without problems. For as was just seen, Husserl assumes the life-world with its intuitive idealities to be the foundation for any idealizations. One should thus expect that one does not really understand the idealizations if one does not know how they originate based on life-worldly experience. And yet Husserl (1984a, p. 249, 2001b, p. 15) also claims that the kind of ideal entities phenomenology describes based on intuitively ideating “differ in principle from those which dominate objective science.” Such a difference in principle, however, would mean that idealization’s foundation in the life-world is irrelevant to understanding idealization. The separation is thus unconvincing. The reason why passively occurring life-worldly types and clear insight into conceptual relations differ is discussed in the next subsection.

As Lohmar (2017, p. 153) (my translation) emphasizes, Husserl differs from Kant in assuming “perception and experience can organize themselves all by themselves” without relying on concepts stemming from the understanding. In Kant (1999, p. A 125–128), the concepts in our scientific judgments about nature are identical to those shaping our experience of nature. Conversely, Husserl separates the concepts in scientific predications and the essences underlying our perceptions of the world. Jansen (2017, p. 143) likewise stresses this difference, assuming even an irreducibility of essences to concepts, because she sees *eide* or essences as ontological entities and concepts as semantic entities.

This interpretation is fitting insofar as Husserl (1939, p. 21, 1973b, p. 27) calls seeing things as this or that in our everyday lives the “pre-predicative experience.” Brudzińska (2017, p. 106) explains this term such that it contains no “veiling” idealities, i.e., no concepts. The term “pre-predicative” is undoubtedly apt insofar as I usually see, for instance, a cell phone as a cell phone without forming a predicative judgment. No subject in contrast to a predicate, no sentence uttered in inner speech, not even the word “cell phone” needs to become aware when we *see* a cell phone *as* such.

Given these anti-mathematical,^9^ anti-conceptual and anti-predicative tendencies in Husserl, one may wonder: How does Husserl propose research on essences and *eide* to be possible?

### 4.1. Eidetic variation as a supposed means to intuitively investigate essences

Husserl’s (1939, pp. 409–443, 1938, pp. 72–87) answer is the method of eidetic variation: One varies the possible appearances of a selected essence, e.g., “table,” “thing,” or “perception,” in imagination. This way, in and through these *imaginative variations*, an *identical essential* structure that is *invariant* throughout the manifold of variations supposedly becomes intuitable. In line with Hume, Husserl (1939, p. 414, 1976, p. 15, 1984b, p. 600) assumes intuiting an essence without a sensory foundation is impossible. However, Husserl (1976, pp. 147–148 1983, pp. 158–160) suggests basing one’s variation on imagination rather than perception, as this way we can easily produce an abundance of variations of a single essence.

When describing eidetic variation, Husserl at times conflates totality and universality. This mistake was discussed above in the context of induction. For instance, Husserl writes eidetic variation requires “an infinite variation in our sense as a foundation.” (Husserl, 1939, p. 423, 1973b, p. 350) If so, one could only say: “*As far as* I have varied this essence in imagination, its eidetic properties are x, y, and z.” His characterization of how to intuit the essence “red” by running through ever more variants makes this mistake: “[A]t each level the red is more red. We anticipate a pure red, a red in pure perfection” (Husserl, 2012, p. 232, my translation). However, the reverse is true: The *same ideality* enables us to see each *different sensory shade of red as the same*, namely as red. The ideal is thus neither the sum nor the mean of all sensory individuals or imaginative variants. It is what allows us to see *each* of them “as *a kind* of x.” In other words: *It is not through the sensory multitude that we get to know the correlating essence. It is through essences that we can distinguish different sensory multitudes*.^10^

Still, eidetic variation is not as ignorant of the employed idealities as Hume’s induction is. Lohmar (2005, p. 86) highlights that its purpose is to bring them to light and clarify them. Moreover, it will also not run into the kinds of malformations that nature presents to us via perception impeding induction. When we vary the essence of a swan, we would, for instance, not imaginatively run through a series of one-winged swans and intuit them just as easily as swans as we would two-winged ones.

However, if all swans we perceived were white, we likely would not imagine black swans in eidetic variation. This is because underlying variations in imagination is what Husserl calls the “apperceptive type.” Lohmar (2005, p. 85) (my translation) summarizes: “The types I have depend on my history of perception; they are by no means universal.” Types are based on previous experiences and what features of the observed instances I happened to notice and passively associate with the type in question (Lohmar, 2005, p. 82). Lohmar (2005, p. 87) furthermore illustrates the cultural relativity of many of our everyday types with a chair as “something to sit,” which takes a different form in Japan and central Europe. Lohmar (2005, p. 87) concedes “that what we think of in a general conception depends on our cultural socialization.” This, however, means that eidetic variation cannot break through the barriers of history, tradition, culture, and language. Lohmar (2005, p. 88) furthermore concedes that non-intuitable essences remain entirely ineffable in eidetic variation.

The types we become aware of by means of eidetic variation are thus unnoticedly shaped by idiosyncratic, cultural, and other prejudices. The problem is that these types may both *contain* associations of *non-essential* features and *lack essential* ones. That is why eidetic variation does not reliably yield insight into essential structures. However, as we can easily run through a manifold of possible variations, it is tempting to misinterpret the underlying types as eidetic or general structures. As a means of becoming aware of one’s idiosyncratic or cultural prejudices, eidetic variation has merit. However, most of Husserl’s investigations into the supposedly transcendental genesis of such structures contribute to psychology, not philosophy (Gutland and Wendt, 2023). Husserl’s analyses help explain how the cultural and historical prejudices that often impede science as a transcultural endeavor arise. They do not, however, reveal eidetic structures as such. That is because in eidetic variation, we cannot reliably distinguish between actual eidetic relations and psychical structures like associations.

An *ex negativo* consequence until this point is that finding essences or idealities must imply an emancipation from what sensory experience provides. After all, sensory perception equally presents individuals having all essential features and those lacking some. Conversely, imaginative variation does offer insight into the types underlying our life-worldly perceptions and beliefs. Yet within these types, essences and psychically rooted structures like associations are intertwined in ways that eidetic variation cannot discern. Therefore, the abstraction process yielding universals must thus be one *away from* sensory experience. Yet what might then be the *positive* source for knowing idealities?

## 5. Hegel on how to discover qualitative idealities

Although Hegel does not use the term “life-world,” he is aware of how most of our everyday beliefs are as firm as they are unfounded. The first step, for him, therefore consists in reflecting on them such that instead of whatever may appear in sensory experience or in imagining, a *different kind of necessity* becomes palpable. Such necessities do appear in mathematical thinking, yet with shortcomings. He points at a shift of conscious awareness which is characteristic for the experience of qualitative idealities.

### 5.1. Overcoming one’s life-worldly prejudices

Hegel (1977, p. 18, 1986c, p. 35) points out that what is life-worldly “familiar, just because it is familiar, is not cognitively understood. The commonest way in which we deceive either ourselves or others about understanding is by assuming something as familiar, and accepting it on that account; […] such knowing never gets anywhere, and it knows not why. Subject and object, God, Nature, Understanding, sensibility, and so on, are uncritically taken for granted as familiar, established as valid, and made into fixed points for starting and stopping.” An example for such a taking for granted of concepts is Kant’s (1998, p. B xvi) first *Critique*, where the concepts “object” and “subject” are presupposed without explanation let alone deduction.

Hegel also describes the life-worldly passivity and arbitrariness in which such psychically rooted prejudices and familiar beliefs appear to us: The “beginnings are immediate, found, or presupposed[…], the form of necessity fails to get its due. Insofar as it aims at satisfying this need, meditative thinking is the thinking that is philosophical in the proper sense” (Hegel, 1991, p. 33/§9). Within the passively appearing beliefs that make up our life-world, we remain unaware which connections are necessary (ideal) and which are contingent like associations. The first step out of this state is to subject this kind of passive knowledge to our thinking, to reflect (meditate) on it. Within our thinking, we can emancipate, in a sense, “purify” our knowledge from the contingencies of sensory and psychically rooted experience.

Hegel describes the different steps of this proceeding for natural science: “It is a great service to discover the empirical numbers of nature, e.g., the distances of the planets from each other; but an infinitely greater service would be to make the empirical quanta disappear by raising them to a *universal form* of quantitative determinations in which they become the moments of *law* or of measure—immortal services which, for instance, Galilei achieved for the motion of falling bodies and Kepler for the movement of the celestial bodies. These men have *proven* the laws they have discovered by showing that the full compass of the singular things of perception conform to them. But a still higher proof of these laws must be demanded—nothing less, namely, than of knowing their quantitative determinations from the qualities or determinate concepts connected in them (such as space and time). Of this kind of proof there is still no trace” (Hegel, 1986d, pp. 406–407, 2010, pp. 297–298).

The first step is thus the establishment of concrete empirical numbers, like the distances between Jupiter and Saturn, via measurements and empirical observation. The second step is to find the respective formulae that—using their universality—allow, e.g., to calculate the distance between Jupiter and Saturn in one year’s time. The third and final step is to *understand* the quantitative formulae *based on thinking the qualitative idealities that are related in them*. The last step is thus not again a way to quantify qualities, but one that proceeds from the discovered quantitative relations toward an understanding of the related qualities and their qualitative relations. Such proceedings are not encountered often, and where they are encountered, the interpretations commonly diverge.^11^ This is, however, hardly surprising, since once we attempt to think purely qualitatively, we are confronted with all our culturally and historically rooted prejudices. This issue shall be picked up further down again.

A pivotal difference between Husserl and Hegel is thus: Husserl assumes that once science idealizes, it betrays its own foundation. Conversely, Hegel sees science’s flaw in idealizing only one-sidedly, namely, quantitatively, but not yet qualitatively. To proceed here, let us look in more detail how Hegel characterizes the emancipation from sensory life-worldly perception that mathematical reasoning offers. Closer characterizing the conscious experience in this kind of thinking later serves as a foundation to contrast how qualitative thinking differs from it.

### 5.2. The one-sided merit and mechanizability of mathematical thinking

Due to its emancipation from the sensory, Hegel (2010, p. 181) sees value in mathematical thinking: “Number is not an object of the senses, and to be occupied with number and numerical combinations is not the business of the senses; such an occupation, therefore, encourages spirit to engage in reflection and the inner work of abstraction, and this is of great, though one-sided, importance.” In mathematical reasoning, therefore, one can experience pure or non-sensory thinking, the content of which is not determined by sensations.^12^ Thus, Hume was right that the propositions are here discoverable “by the mere operations of thought.”

Hegel explains mathematics’ one-sidedness in that “since the basis of number is only an external, thoughtless difference, the occupation proceeds without a concept, mechanically” (Hegel, 2010, p. 181). The externality was already illustrated with how quantification transforms a contradiction, an angel, and Jupiter each into an indifferent “one.” Afterward, because we abstracted from any qualitative connections that would normally prevent this, we can combine the “one” at will in line with some arithmetical procedure. That this kind of thinking occurs nearly entirely self-external has two further effects.

Hegel already mentioned the first: Mathematical thinking can be *mechanized*, or, in general, computerized. Notably, Husserl concurs: A mathematical “solution can be obtained in a purely mechanical fashion. This happens in that one substitutes the names for the concepts, and then by means of the systematic of names and a purely external process, derives names from names[…;] calculating […] is not an activity with concepts, but rather with signs” (Husserl, 1970a, pp. 239–240, 2003, pp. 253–254). Husserl (1975, p. 79, 2001a, p. 50) therefore stresses that computers do not think: The ways in which the results “spring forth is regulated by natural laws which accord with the demands of the arithmetical propositions which fix their meanings. No one, however, who wants to give a physical explanation of the machine’s procedures, will appeal to arithmetical instead of mechanical laws. The machine is no thought-machine, it understands neither itself nor the meaning of its performances.” In other words: While we do mathematics, we *experience* conceptual necessities *in consciousness*. Computers have no such experience. They are built such that *instead of experiencing concepts*, they connect *symbols* in accordance with *natural laws*. This connecting is *directly* explainable by laws of nature (namely mechanical, electrical, maybe chemical laws).

A computer’s output is thus no concept, but a symbol. This output symbol is directly processed and generated not by arithmetic laws, but by physical ones. If a computer is broken, all its parts and processes still adhere to natural laws. But the way these parts then function, while still in accordance with natural laws, no longer indirectly (symbolically) adheres to arithmetic laws. However, when seeing computer-generated output symbols, we are usually able to go beyond the mere sign and *understand* its meaning: We can *think* the respective concept. We should not, however, assume the computer experiences these symbols alongside the respective concepts like we do. Probably one of the greatest misunderstandings of human thinking is the belief that it resembles a computer’s processing and can be modeled algorithmically.

The second consequence of the self-externality of mathematical thinking is noticeable within the thinking experience itself: The thinker has a high degree of freedom and control. The initial impulse *which* thoughts to connect *and how* depends entirely on her. For instance, after quantifying Jupiter, an Angel, and a contradiction, we are free to add them all up to three, or add up only two and subtract the third, or divide one by the product of the two others, etc. Hegel (1977, p. 26) explains: “In a non-actual element like this there is only a truth of the same sort, i.e., rigid, dead propositions. We can stop at any one of them; the next one starts afresh on its own account, without the first having moved itself on to the next, and without any necessary connection arising through the nature of the thing itself. […] For what is lifeless, since it does not move of itself, does not get as far as the distinctions of essence, as far as essential opposition or inequality, and therefore does not make the transition of one opposite into its opposite, does not attain to qualitative, immanent motion or self-movement.”

Hegel here metaphorically calls mathematics “dead” due to the lack of self-movement of the contemplated content. As in mathematical thinking the “one” is cut from its qualitative content, this qualitative content can no longer be our “guide” to whatever is qualitatively related to it. Consequently, the impulse to connect it with other thoughts must come from us: *We* control with which other “one” to relate it and also whether we add, subtract, or multiply them. Nothing “happens” in mathematical thinking if we do not initiate such an impulse. Only afterward, and depending on what we try, we encounter necessities^13^ within this thinking. Yet we come across them by so to speak “bumping into” them. They prove to be “obstacles” of what we can do, “guiderails” of where we can go and what follows if we go through with our initial impulse. Notably, we *experience* that we cannot do entirely as we please. Nevertheless, for instance when trying to prove a theorem, we may still freely make a plethora of possible choices.^14^

These conceptual necessities both constrain and guide us in our thinking experience, indirectly helping us to proceed to the solution. That they have no self-movement is the reason why they can be symbolically “outsourced” to laws of natural causality. Just like the result of a calculation depends on what we decided to calculate, the computer’s output as a physical effect is shaped by the input as a physical cause. The rigidity and non-self-movement of arithmetic laws is the reason why they can be symbolized in something as rigid as natural causality. Our freedom, however, cannot be transferred this way. We still must decide what the computer is to compute.^15^

In sum, it would be wrong to conceive of the necessities we encounter in mathematical thinking experience as in some way sensory. And neither do they stem from cultural or historical biases. They are actual thinking experiences—we *encounter* them *experientially*. Yet how does mathematical thinking differ from qualitative thinking?

### 5.3. The shift in conscious experience when thinking qualitatively

Hegel assumes that even though quantity occurs within it, logical thinking outstrips mathematical thinking. Therefore, he fiercely opposed attempts like Leibniz’s to mathematicise logic (Hegel, 2010, pp. 607, 544). That is why for Hegel, logic entails quantitative and qualitative thinking. He writes that within pure logic “thoughts are grasped in such a way that they have no content other than one that belongs to thinking itself, and is brought forth by thinking. So these thoughts are *pure* thoughts” (Hegel, 1991, p. 58/§24). Herein lies the emancipation from the sensory: Both perception’s malformations and prejudice-based imaginations need no longer distract us when we meditate purely on thought contents themselves. Only based on what we find in this kind of thinking can we, e.g., identify malformations as such, discover essential relations we do not associate and realize that our associations are non-essential.

Yet the description thus far would also be adequate to mathematical thinking. What separates qualitative thinking from it becomes evident in Hegel’s further characterization: “When I think, I give up my subjective particularity, sink myself in the matter, let thought follow its own course; and I think badly whenever I add something of my own.” I must thus give up the freedom I still have in mathematical thinking. Instead of myself being the motor of what I wish to combine and in what way, I actively *observe* how my thoughts unfold in and out of themselves.

The possibility to “drop out of” this observing is much higher than in mathematical thinking. Relatedly, the “adding something of my own” refers to idiosyncratic associations, passive syntheses based on life-worldly types, cultural beliefs, traditional convictions, and so on. They amount to what Hegel calls the “subjective particularity.” All those influences are ready to distract me. They occur with the same passivity as in everyday perceptions, and they tempt me to *judge* based on them rather than continue to *think* and therein *observe* how the thought evolves in and out of itself. If I do not keep them at bay or fail to notice how they influence me, these familiarities likely lead me astray.

Thus, whenever attempting to find out how one thought relates to another qualitatively, we quite literally *fight ourselves*. A much lesser self-discipline, but still some, is needed when performing mathematical thinking. After all, doing mathematics, i.e., solving an equation, is not guaranteed to succeed. Even the most outstanding mathematicians err at times. A mathematician who can self-reflect on how she does her work will know the difference between judging step by step based on actual experience versus based on guessing or simply drawing on memory. An example: One may remember that 12 × 12 = 144. Nevertheless, one can also reaffirm this by entering actual mathematical thinking. The first kind of judging would be one where I draw on what Hegel calls my “subjective particularity.” However, only the second one deserves to be called “thinking” over and above mere “judging,” as it involves *experiencing* the necessity of the result. Naturally, judging, e.g., based on memory, is not necessarily wrong.

Hegel speaks of “life” in qualitative thinking because of the “self-movement” of thoughts therein. In this thinking, although I initially can still freely choose a thought to focus on, this thought, then, leads me to a different thought. It, in a sense, becomes this other thought, illustrating through this movement how both thoughts are related. Then the second thought leads to a third or moves back to the first, revealing a hitherto unnoticed qualitative relation. There is an immanent rhythm guiding the course of this thinking. It evolves akin to an organism. Self-movement of the thought and growth of our resulting knowledge are the two facets it shares with life.

Mathematical thinking does not have such properties. Otherwise, it would not be mechanizable. A certain quantity like 7 does not, through self-movement, become 8. It was shown how we must thoroughly abstract from thinking any qualities to handle quantities properly. Instead of experiencing qualitative necessities, *we* initiate combinations within the mathematical possibilities, experiencing the respective quantitative necessities as we move along.

We *lose* this control once we manage to think qualitatively. Then we are no longer the ones who connect a thought with the next, but the thoughts *relate themselves*. Experiencing this lack of control and how, instead, something else “takes over” *in one’s own consciousness* can at first be startling. Being in control as we are when doing mathematics is certainly at first more comfortable. That is why for many first experiencing the life Hegel describes does not at all feel comfortable. However, the price one pays for remaining in control within mathematical thinking is absence of guidance by qualities.

Johann Gottlieb Fichte’s characterization of qualitative thinking is remarkably similar. Fichte (1982, p. 30) emphasizes that in it we are not dealing with “a lifeless concept, passively exposed to its inquiry merely […], but a living and active thing which engenders insights from and through itself, and which the philosopher merely contemplates. [H…]ow the object manifests is not his affair, but that of the object itself, and he would be operating directly counter to his own aim if he did not leave it to itself, and sought to intervene in the development of the phenomenon.” He contrasts this kind of thinking with the typical philosophical “system-makers,” who “proceed from some concept or other; without caring in the least where they got it from, or whence they have concocted it, they analyze it, combine it with others to whose origin they are equally indifferent. [The philosopher] is fashioning an artifact. In the object of his labors he reckons only upon the matter, not upon an inner, self-active force thereof. Before he goes to work, this inner force must already have been killed, or it would offer resistance to his efforts” (Fichte, 1982, pp. 29–30). Fichte’s characterization here resembles Hegel’s right down to the metaphors of life and death.

In sum, human reasoning can take different forms. The first, life-worldly one, operates with whatever types have already been established and—sometimes more actively, sometimes more passively—combines them with other such previously established types. One judges, not necessarily using language, but one usually does not make thinking experience one’s anchor point for making these judgments. The thinker herself or her cultural, social, traditional, etc. biases are the driving force for the how and what of the connecting. In mathematical thinking, the thinker controls which concepts she thinks about and how she attempts to connect them with others. We experientially encounter conceptual necessities, but no connection comes about through the self-movement of the conceptual content we think. Lastly in qualitative thinking, the freedom is reduced to the choice of thought to begin the reflection with. After initiating this reflection and focusing on the thought, the thinker gives up her freedom to control the development and instead observes how the thought unfolds out of itself and into others. The thinker, “instead of being the arbitrarily moving principle of the content, [chooses] to sink this freedom in the content, letting it move spontaneously of its own nature, by the self as its own self, and then to contemplate this movement” (Hegel, 1977, p. 36).

After offering this brief characterization of qualitative thinking, it is now time to address some of the many concerns that thus far prevent it from being widely accepted.

### 5.4. Critical reflection

A first concern is that while Hegel and Fichte similarly characterize qualitative thinking, their philosophical systems nonetheless differ. How to account for this difference? Are the insights within qualitative thinking different for each person? If so, how could it be scientific? If it is scientific and thus the same for everyone, would not it imply that those who fail at it would have to blindly believe those who claim to be able to perform it? Would that not lead to an impoverishment of cultural diversity such that we would end up with a scientific monopoly instead of a pluralism of rich and historically grown perspectives?

Trains of thought like these show how a possible scientific insight into qualitative essential relations soon turns political. The fears underlying these questions need to be taken seriously. After all, did Foucault not show how truth in science is prone to power dynamics that frequently undermine rather than foster its discovery and acceptance? And yet, even Foucault (1974, p. 24) refrained from a thorough relativism, for instance, when stating Mendel told the truth albeit the discourse of his time rejected it. Against the worry that scientific truth is a façade for political power, it must be emphasized that the point of qualitative thinking as portrayed here is not that one should let others dictate what is true. Rather, it was to inspire confidence that in principle *everyone in their own thinking can experience* essential qualitative relations as *they* are. Intersubjective exchange is but one of several ways to find a truth. At best it helps find the truth, but it cannot substitute one’s own experience of veracity.

Due to an article’s brevity, some of the other concerns are best countered with other questions. For example: Does the word ‘truth’ remain meaningful and valuable if there are as many truths as there are traditions, languages, and cultures? Is it only negative if the search for truth ultimately unites people from all cultures and traditions rather than discriminating against them based on their backgrounds? And with regard to historically grown perspectives, Scheler (1976) offered a noteworthy suggestion of how the historically accumulated knowledge of the different cultures and traditions could be construed as complementing each other.

For sure, this will not ease everyone’s reservations or cease their doubts. Especially the phenomenological tradition has rejected a thinking on which everyone can agree, irrespective of their culture and tradition. Husserl (1962, p. 396) himself sometimes treats scientific theories as if they were merely cultural constructs. He thereby inspired relativisms, for instance in Heidegger, Gadamer, and Derrida, who became inspirational figures for postmodernism (McGee and Warms, 2008, p. 536), a movement questioning all objectivity.

It is common to find phenomenologists appealing to factors akin to the life-world as both an ineffable and unsurmountable foundation that relativizes all we can ever know. Heidegger (1993, p. 29) points at tradition and calls it naive if someone assumes a fresh beginning in philosophy to be possible. Gadamer (2010, p. 361) rejects Hegel’s hint at an experience of pure thinking, instead maintaining to “be situated within a tradition does not limit the freedom of knowledge but makes it possible” (Gadamer, 2004, p. 354). Merleau-Ponty invokes the lived body as such a primordial foundation. Even when it comes to abstract space, as the geometer conceives it, Merleau-Ponty (2002, p. 117) maintains that “there would be no space at all for me if I had no body.” Waldenfels (2006, p. 109) claims it to be impossible to objectively compare cultures because we belong to one from the outset. Stähler (2003, p. 239) holds that in the face of history, it is hubris to believe that true knowledge is possible, as Hegel did. Whichever of such factors (or a combination) one favors, the consequence is that it undermines our attempts to achieve true knowledge and knowledge of universal structures.

However, one must distinguish between such factors and *our* judgment about them. Suppose at some point in our life, we become convinced that our tradition opaquely influences all our judgments. Because we cannot know where tradition misleads our judging, our judgments would become untrustworthy. Then, however, the same would apply to our initial judgment on tradition leading us astray. We could only trust our judgment on tradition if we knew that this judgment is *not misguided* by tradition. Yet precisely this kind of knowledge would be impossible if this judgment were correct. Therefore, judgments like these are performative self-contradictions. They judge to be impossible what they presuppose as true. The same ratio applies to any of the named factors one might choose here.

And yet, not only those who oppose the existence of universal truths, but also those who propose them, often jump to conclusions. A more intricate analysis of the possible shifts in conscious experience is thus required. Particularly, one needs to learn to distinguish between the acts of judging and thinking. Otherwise, one is unable to distinguish the passively occurring psychical prejudices from the self-movements of thoughts.

Judging, because it is a psychical process, is prone to the mentioned factors. And we cannot form a belief without judging. What we experience in thinking neither causes nor otherwise forces us to also judge it to be as experienced. This likely is the reason why Hegel’s and Fichte’s systems differ. In our everyday judgments, such life-worldly prejudices usually remain effective even after we become aware of their contingent and psychically rooted constitution. Unless we encounter a viable alternative in thinking or perceiving and decide to consciously judge in accordance with it, every now and then remembering this new insight, we hardly stand a chance to alter our prejudices.

In many ways, the endeavor to think purely is thus preceded and impeded by the psychical forces embedded within our life-world. Without our life-world, however, we could not even master a single day in our everyday life. Thus, the life-world is not simply something bad. Epistemically opaque as it is in its passivity, it has an indispensable pragmatic value for our everyday lives.^16^ Nevertheless, if we wish to attain ethical responsibility instead of being passively driven, we need to become aware our life-worldly preconceptions, reflect on them, and correct them where required. One way to do this is to enter qualitative thinking. Its experience provides either viable corrections or consciously understood confirmations of the passively intruding life-worldly prejudices. Based on it, we can choose to overcome our respective prejudices. Naturally, within thinking, we can do so only for our judgments on *essential* relations. Sensory perception remains the corrective of our life-worldly beliefs about *individual facts*.

The point here thus was not to claim that Hegel, Fichte, or another thinker “got it all right.” Instead, it was to remind of the possibility of an experience in which everyone could for themselves find out how thoughts interrelate within thinking.^17^ Needless to say, given the sheer richness of the ideal content of our world, hopes as well as fears that one could quickly lay it out in its entirety once and for all are equally unfounded.

## 6. Summary and synthesis

This article drew on the philosophies of Husserl and Hegel to analyze the quantitative proceeding in science and to remind us that a qualitative thinking might be possible. It is now time to offer a synthesis of the main points. After that, an outlook is in order, considering some more recent developments in science that were not available in Hegel’s and Husserl’s time.

In order to quantify something, we must abstract from all its qualitative features and relations. We then enter a pure and clear thinking in which everyone can contribute and cooperate regardless of their traditional or cultural background. The abstraction is so thorough that it leaves behind what is at stake in most cultural or historical controversies. However, the abstracted qualities remain detached from mathematical thinking. We cannot mathematically establish units, measure, or determine whether $E=m\;c^{2}$ or $E=\sqrt[{m}]{c}$ is true. Knowledge beyond mathematics has been acquired in science mostly through empirical observation and hypothetical reasoning. But a way of thinking that is as rigorous, clear, and interculturally uncontroversial as mathematical thinking, yet able to operate directly with qualities, is still a desideratum.

Historically, philosophy and physics have “cooperated” in denying the qualities we experience by excluding them from scientific objectivity. Physics deconstructed the objectivity of the secondary qualities as “merely subjective.” Hume then claimed that quantitative idealities are discoverable by the mere operations of thought, while setting up empirical standards for qualitative idealities that quantitative idealities would fail. As a result, the qualities populating our life-worlds were mostly excluded from rigorous science. Yet Husserl showed how our life-worldly experience, including its qualities, underlies the way science is practiced even though scientific theory denies most life-worldly aspects.

If science wants to overcome its naïve use of the life-world and its qualities, it must critically reflect on the way it relies on it. Husserl and Hegel offered different ways of doing this. Husserl provided methodological means for becoming aware of the structures of the life-world. He also offered psychological investigations that help to understand how the contingent life-worldly associations and passive syntheses come about. One important result is: It is almost impossible for two people to have exactly the same life-world.

However, Husserl failed to see that his supposedly transcendental investigations on the genesis of consciousness are contributions to psychology, not epistemology (Gutland and Wendt, 2023, pp. 112–115). As long as we stay within life-worldly reasoning, we cannot find out how two ideal qualities are *ideally* related. This cannot be achieved by becoming aware of how we or other subjectivities happen to life-worldly associate qualities, nor by becoming aware of how these associations are genetically constituted.

Such a positive source of actually experiencing qualitative idealities and their relations is given in qualitative thinking as characterized by Hegel and Fichte. The characteristic shift of consciousness going along with it was contrasted with the conscious experience during life-worldly associating and mathematical thinking. Like life-worldly associating, qualitative thinking contains qualitative ideal contents, but does not connect them based on contingent psychical forces. Like mathematical thinking, qualitative thinking is clear and pure, but instead of oneself controlling the how and what of combining, one allows the thought to unfold itself. The shift of consciousness here is thus such that one keeps up the active attention but becomes a passive observer of how one’s thinking spins itself forth from one thought to the next.

If qualitative thinking is to be established scientifically, combining the insights of Husserl and Hegel is a viable starting point. One would have to develop and contrast them further. If one only consults Husserl, one easily falls prey to the mentioned relativisms, then claiming all our thinking is ineffably prone to factors like tradition, culture, language, or history. If one only consults Hegel, one easily underestimates the psychical force of the life-world, believing one is thinking purely and selflessly, while in reality, one’s life-word passively and unnoticedly shapes one’s beliefs. What is required, instead, is learning to distinguish life-worldly judging from qualitative thinking *based on their different characteristics in conscious experience*. Otherwise, one will conflate life-worldly passive syntheses and the kind of passivity encountered in qualitative thinking. One will mistake one’s psychically rooted cultural prejudices to be the self-movement of thoughts and vice versa. Among other things, it is important to distinguish the different *act types* of pure thinking and judging and learn to recognize their characteristics (Gutland, 2021). In short, one must not only be aware of the content that one connects, but one must extend one’s awareness to the *How* of that connecting.

Yet how would qualitative thinking impact science and integrate with its quantifying and measuring?—Qualitative thinking deepens our understanding of the elementary concepts in science as well as their relations. Hegel (1986b, pp. 41–47/§§ 254–256), for instance, faults the quantitative approach for handling space’s three dimensions—length, height, and width—as entirely interchangeable. There is nothing about the *x*-axis that prevents us from instead calling it the y-axis and vice versa. Hegel believes this is so because few people observe how space’s three dimensions develop out of one another in qualitative thinking. Instead, they are presupposed and then only quantitatively, i.e., abstracting from their qualities, related in equations. The same is the case in other elements of equations. Even simple ones like Newton’s f=ma are quantitative expressions of qualitative relations. The more we learn to think the qualities involved and how they relate qualitatively to other qualities, the more we would ideally or essentially understand the Why.^18^ Qualitative thinking thus does not replace or invalidate the quantitative relations, it would deepen our understanding of the related qualities as such.

Using quantitative modeling in combination with experimental data gathering, one can only establish *that* certain formulae adequately model reality. Without thinking through the qualitative elements and relations connected therein, we mostly fail to understand the *Why* of them. Even quantum physics, where stochastic probabilities replaced Newton’s belief in necessary determination, is no exception. After all, knowing that an event occurs with a certain probability differs from understanding why this is so based on reflecting on the essential nature of the involved qualities.

Table 1 provides an overview of the respective advantages and disadvantages of the life-world, scientific objectivity, Husserl’s phenomenology, and qualitative thinking.

**Table 1:** An overview of the subject areas and their differences as covered here.

	The life-world	Scientific objectivity	Husserl’s phenomenology	Qualitative thinking
Advantage	Populated with qualitative content, culturally and historically rich.	Emancipates itself from life-worldly associating insofar as it sticks to a quantifying methodology. Applying this methodology to the world, science uncovered vast amounts of reliable, objective, and valid quantifications of the qualities structuring reality.	Provides psychological methodological means to become aware of life-worldly structures and investigate their genesis.	Provides direct experiential access to essential qualitative relations. This allows us to deepen our understanding of the quantitative relations found thus far and discorver new ones.
Disadvantage	The connections of its content vary from person to person, as they are brought about mostly passively and associatively.	Its quantitative emphasis detaches it methodologically from directly investigating qualities and qualitative relations. The ways it nonetheless relates to qualities are: empirical observation, hypothetical reasoning, and the situatedness of scientific practice within the scientist’s life-world. These dependencies on qualities, especially the last one, frequently are used without much critical reflection.	Cannot methodologically distinguish between psychological associations and essential relations.	Hard to attain and, without clear and critical awareness of the difference between the act types of judging and thinking, easily confused with life-worldly passivity.

There certainly are scientists who already today look through and beyond mathematics such that they form some notion of the *Why*. This allows them to have valuable hunches and intuitions, inspiring discoveries. However, because of the current quantitative emphasis, they cannot convey their insight in an academically acceptable manner. This article is written not least in the hope that such knowledge can, in the future, be shared in scientifically accepted ways.

Today, the emphasis on quantity in science has come full circle: Quantification, instead of being used by scientists, is now being used on them. Lazebnik (2015, p. 1599) describes a worrying trend in science: “reputation based on discovery is no longer the currency.” Although Lazebnik (2015, p. 1599) mentions several causes, he also mentions how scientists are now frequently assessed by “the number of papers published, the number of citations, citation indexes, impact factors, formulas to calculate their relative values.” Such criteria are not *per se* wrong, but they are one-sided. At first, they seem convenient: Anyone can check article numbers and citations, as it is both easier and quicker than reading them and objectively assessing their quality. But this convenience is due to the abstraction inherent in quantifying. Like Jupiter, a contradiction, and an angel, three published articles are three, whatever their quality is. And this is where the one-sided focus on quantity begins to hollow out academic work. For—within such assessment criteria—someone with 10 mediocre, repetitive articles is ‘better’ than someone with five original ones that tackle and solve difficult problems. Practices like being hired or fired based on the number of one’s publications are thus an example of where the ability to instead find and establish objective criteria for directly assessing quality would warrant science’s own quality does not decline. Relatedly, another hope out of which this article was written is that it helps ensure scientists can again, without worry, delve into difficult and time-consuming problems, knowing that their results will be evaluated beyond being a mere “one.”

## 7. Outlook: scientific developments since Husserl’s and Hegel’s days

I am grateful to one of the reviewers suggesting to also relate the key finding here to how today’s science has developed since Hegel’s and Husserl’s days. However, since these two philosophers are its focus, this article had first to discuss ‘their’ respective science. Now that their standpoints have been discussed, what changes in light of some newer developments may be briefly considered.

For a long time, a belief in natural science was that the principles explaining all phenomena must be found on the smallest scale. All processes on larger scales would then be explainable by these smallest scale processes, i.e., reducible to them. To be precise, this belief has been predominant in physics, while chemistry proceeded early on to assign to certain structures a non-reducible or “autonomous meaning” (Di Paola et al., 2013, p. 1598). This is even in line with Hegel (2010, p. 646), who called “the chemical object […] a self-subsistent totality.” The reductionist belief, however, had quite some discursive force in the sense of Foucault. For instance, “emergent organized behavior” on the mesoscopic scale was “accepted as true only after repeated confrontations with experiment left no alternative” (Laughlin et al., 2000, p. 32). By now, in many scientists, the belief in a theory of everything, i.e., “a set of equations capable of describing all phenomena” (Laughlin and Pines, 2000, p. 28), gave way to the realization that different principles rule phenomena on different scales.

Meanwhile, the ideal of infinitely accurate measurements that Husserl mentions was undermined by both Heisenberg’s uncertainty principle and the realization that, for instance, calculating interaction of more than 10 balls on a billiard table is impracticable (Weaver, 2004, p. 67). And yet, when one abstracts from measuring all details, one may take a step back and use stochastics to compute probable developments. As Weaver (2004, p. 68) elaborates, within such approaches, which are also successfully used by life insurances, the “method applies with increasing precision when the number of variables increases.” Here, higher-order principles emerge that are again not predictable by knowing the principles on smaller scales, i.e., these higher order principles are not reducible.

Nevertheless, within empirical reality the belief might persist that there could be some as yet unobserved link confirming reductionism. Yet emergence and complexity appear even in mathematics. When used to model population growth, the logistic function mentioned above may lead to emergent phenomena like bifurcations and self-similarity (Richter and Rost, 2002, pp. 9–24). Another example would be John Horton Conway’s mathematical *Game of Life* that, on higher scales, shows patterns rendering impossible to deduce the simple mathematical principles that govern them (Richter and Rost, 2002, p. 40). That emergence also occurs in mathematical contexts rules out denying it by appealing to insufficient empirical observation.

The discovery and establishment of emergence and complexity are also relevant for our conscious life-worldly experience, for they may re-establish aspects of our conscious phenomenal experience as scientifically objective. For instance, Giuliani et al. (2014, p. 1) explain that the reductive approach “considers biological systems having a strictly hierarchical architecture going from molecular to whole organism level and in which the ultimate causative layer is the most microscopic one, i.e., the molecular level (genes).” Consequently, the phenotypes that appear as wholes in our conscious experience would be subjective illusions. Objectively, instead of wholes, they would be manifolds of molecular processes causally orchestrated by—and thus reducible to—laws on the genotype level. Yet Heckman (1990, p. 782), for instance, showed that “shape changes […] have physiological significance in cells.” Observations like these, combined with emergence and complexity, might objectively rehabilitate the perception of wholes as they appear in our conscious experience.

The receding reductionism incited a “surge of interest in graph-theoretical and, in general, network-based approaches in both physics and biology” (Di Paola et al., 2013, p. 1598). Such approaches have been successfully used to find new drugs, where these more systemic approaches prove more efficient (Csermely et al., 2013). Also, in drug development, approaches like principal component analysis (PCA) overcome the need to—before one even begins analyzing a set of data—assume certain variables to find the other variables based on these assumed ones. Instead, purely by analyzing the data set, one can identify the “hidden independent factors modulating a given set of observed variables” (Giuliani, 2017, p. 1070). Insofar as cultural biases can make one assume certain variables rather than others, such biases can thus be overcome.

In Husserl’s and Hegel’s time, approaches like these were unavailable not least due to the sheer amount of computational data processing they require. Approaches like these have considerably enriched our scientific understanding of the complexities that underlie and govern our world. They overcame the deterministic and reductionist “if-then” kind of thinking and made emergent relational or systemic structures scientifically accessible and acceptable. As noted above, they may even reconcile conscious phenomenal experience with scientific objective reality, which would be a major scientific breakthrough.

Yet the procedure is still to quantify these qualitative structures. In chemistry, the goal is “to derive mathematical descriptors of molecular structures” (Di Paola et al., 2013, p. 1598). The network approach is described as “a quantitative framework” (Di Paola and Giuliani, 2015, p. 47). The graphs used in network approaches are described as “a mathematical object used to model complex structures” (Giuliani et al., 2014, p. 2). Likewise, “PCA can be thought as the fitting of an *n*-dimensional ellipsoid to the data, where each axis of the ellipsoid represents a principal component” (Giuliani, 2017, p. 1075). Thus, approaches like these should not be confused with the kind of qualitative thinking outlined above.

However, just like Kepler’s or Newton’s laws, the quantified structures uncovered by these approaches provide a significant orientation for qualitative thinking. And not only that, for—within these new approaches—we can identify steps that would benefit from qualitative thinking. For instance, Csermely et al. (2013, pp. 337, 342) mention several times the difficulties in defining nodes and edges of networks. Giuliani (2017, pp. 1070–1071) mentions the need to give names to components that result from rotating and collapsing a data set within PCA. Here, a qualitative understanding of the involved concepts and their relations would prove very valuable. Examples like these thus show the complementary and cooperative potential between these newer approaches and qualitative thinking as outlined here. Ideally, there would be an interplay and mutual fostering between observation, quantification and qualification.

## Data availability statement

The original contributions presented in the study are included in the article/supplementary material, further inquiries can be directed to the corresponding author.

## Author contributions

The author confirms being the sole contributor of this work and has approved it for publication.

## Footnotes

^1^The restriction to quantity has its problems, as will become clear when discussing geometric idealities. However, it helps to portray the benefits and issues with quantification much more clearly.

^2^The quality of the English translations of Husserl is frequently called into question. Where available, I quote the English translation but also indicate the German original’s pages.

^3^This difference between mathematical correctness and actuality is reminiscent of the difference between validity and soundness in logic.

^4^Quine (1951, p. 41) parallels such hypothetical elements with believing in gods: “[I]n point of epistemological footing the physical objects and the gods differ only in degree and not in kind. Both sorts of entities enter our conception only as cultural posits.”

^5^See Urry et al. (2021, p. 1199) figure 53.10 (a) for a graph with similar characteristics as described here while contrasting the logistic function with actual measurements in a growing *Paramecium* population.

^6^Biologists are aware of one deviation that I do not mean here: “The logistic model assumes that populations adjust instantaneously to growth and approach carrying capacity smoothly. In reality, there is often a delay before the negative effects of an increasing population are realized.” (Urry et al., 2021, p. 1199) Such a delay could be modeled using a different function. What I mean here, however, are the inevitable deviations between the ideal curve and actual measurements even if populations adjusted instantaneously.

^7^Already Democritus (Diels and Kranz, 1972, pp. 139, 166, 168), believing only atoms and the void to be real, concluded that colors and tastes are not. Aristotle (2016, pp. 34–35/418a) noted that some qualities, like color and tones, are “exclusive to an individual sense,” while he calls qualities like “motion, rest, number, shape, and magnitude” ‘common objects,’ “since these sorts of objects are exclusive to no one sense but are, rather, common to them all.” After all, we can get to know an object’s shape through touch *and* vision, but we cannot touch an object’s color. Observations like these gave rise to the notion that specific sense qualities are more subjective, potentially only being effects of specific sense organs rather than objective properties of perceived objects. This is precisely what Johannes Müller claimed. Müller (1844, pp. 667–668) observed that an identical stimulus brings about different sense qualities in other sense organs. He concluded that the specific sense qualities are relative to the respective organ and do not correlate with anything objective. Unfortunately, delving into the logical fallacies of this line of reasoning would lead astray here.

^8^The general idea underlying induction—that experience of individuals is the yardstick for accepting or rejecting idealizations—is still present in Popper’s (2002, pp. 3–10) attempt to replace induction with deduction.

^9^Besides refusing to understand phenomenological essences mathematically (Husserl, 1984a, p. 249, 2001b, p. 15), Husserl (1976, p. 127) also excludes formal logic and Leibniz’s *mathesis universalis* from phenomenology.

^10^Some reject conceiving eidetic variation as akin to Hume’s induction: Lohmar (2005, pp. 79–80) because he maintains the current variation already includes future ones in the idea of an ‘I can.’ Brudzińska (2017, p. 112) and Jansen (2017, p. 145) because its imaginative freedom liberates eidetic variation from induction’s dependency on an empirical and thus factual manifold. I nonetheless maintain that the problem of induction exists as long as one binds intuiting essences to sensory variation. Such varying is but one of many possibilities to intuit essences. Scheler (2018, p. 64) stressed that ideation is possible based on just one example. Fichte, Schelling, and Hegel assumed it to be possible in pure thinking, i.e., independent of concurring sensory experience.

^11^Some notable attempts at thinking through scientific observations philosophically do exist, e.g., in Scheler’s (2018) and Plessner’s (1975) attempts to establish philosophical anthropology.

^12^In the same volume this article is written for, Ziegler and Weger (2023) offer a description of the experience when thinking through a mathematical proof.

^13^Since these necessities still depend on the chosen set of axioms, they are conditioned necessities.

^14^Hegel (1986c, pp. 42–44) assumes this arbitrariness even for geometrical theorems. That, however, might go too far since geometrical idealities are still qualitative unless arithmetized.

^15^Exploring how far the recent developments in artificial intelligence and quantum computers changed this would lead too far here.

^16^Notably, more recent attempts to model human decision-making based on quantitative functions seek ways to include tacit knowledge, which would be roughly equivalent to life-worldly knowledge (Bizzarri et al., 2022).

^17^Throughout this text, thinking is described as an experience. Within the cognitive phenomenology debate (Bayne and Montague, 2011), some reject this. Shields (2011, p. 233) mentions a reason for this: If thinking were an experience, then instead of being part of a functionalist solution, it would become part of the so-called ‘hard problem of consciousness’ (Shear, 1999). See Gutland (2018, 2021) for attempts to closer characterize the experience of thinking and to answer whether this experience has a phenomenal character.

^18^That empirical reality, e.g., in the form of measurements, may slightly deviate from essentiality should have become clear by now.

## References

[ref1] Aristotle (2016). De Anima. ed. Shields C. (Oxford: Oxford University Press).

[ref2] Bayne T. Montague M. (2011). Cognitive Phenomenology. Oxford: Oxford University Press.

[ref3] Bizzarri F. Giuliani A. Mocenni C. (2022). Awareness: an empirical model. Front. Psychol. 13:933183. doi: 10.3389/fpsyg.2022.933183, PMID: 36571049 PMC9780538

[ref4] Brown H. I. (1992). Direct realism, indirect realism, and epistemology. Philos. Phenomenol. Res. 52, 341–363. doi: 10.2307/2107939

[ref5] Brudzińska J. (2017). “Erfahrung und Urteil” in Husserl-Handbuch: Leben—Werk—Wirkung. eds. Luft S. Wehrle M. (Stuttgart: J.B. Metzler), 104–113.

[ref6] Carrier M. (2009). Wissenschaft im Wandel: Ziele, Maßstäbe, Nützlichkeit. Info. Philos. 3, 16–25.

[ref7] Csermely P. Korcsmáros T. Kiss H. J. M. London G. Nussinov R. (2013). Structure and dynamics of molecular networks: a novel paradigm of drug discovery: a comprehensive review. Pharmacol. Ther. 138, 333–408. doi: 10.1016/j.pharmthera.2013.01.016, PMID: 23384594 PMC3647006

[ref8] Spinoza B. de. (2018). Spinoza: Ethics: Proved in Geometrical Order. (ed.) Kisner M. J. Cambridge: Cambridge University Press. Trans. Silverthorne M. Kisner M. J.

[ref9] Descartes R. (2008). Meditations on First Philosophy. With Selections From the Objections and Replies. ed. Moriarty M. (Oxford: Oxford University Press)

[ref10] Di Paola L. De Ruvo M. Paci P. Santoni D. Giuliani A. (2013). Protein contact networks: an emerging paradigm in chemistry. Chem. Rev. 113, 1598–1613. doi: 10.1021/cr3002356, PMID: 23186336

[ref11] Di Paola L. Giuliani A. (2015). Protein contact network topology: a natural language for allostery. Current Opinion in Structural Biology. 31, 43–48. doi: 10.1016/j.sbi.2015.03.001, PMID: 25796032

[ref12] Diels H. Kranz W. (1972). Die Fragmente der Vorsokratiker. Zweiter Band. 16th Edn Hildesheim: Weidmann.

[ref13] Fichte J. G. (1982). The Science of Knowledge: With the First and Second Introductions. eds. Heath P. Lachs J. (Cambridge: Cambridge University Press)

[ref14] Foucault M. (1974). Die Ordnung des Diskurses, Inauguralvorlesung am Collège de France—2. Dezember 1970. ed. Seiter W. (München: Carl Hanser).

[ref15] Gadamer H.-G. (2004). Truth and Method. eds. Weinsheimer J. Marshall D. G.. 2nd ed (London: Continuum).

[ref16] Gadamer H.-G. (2010). Wahrheit und Methode. Grundzüge einer Philosophischen Hermeneutik. 7th Edn Tübingen: Mohr Siebeck.

[ref17] Galilei G. (1957). “The Assayer” in Discoveries and Opinions of Galileo. ed. Drake S. (New York: Doubleday & Co.), 231–280.

[ref18] Giancoli D. C. (2014). Physics for Scientists & Engineers With Modern Physics (4th). Essex: Pearson.

[ref19] Giuliani A. (2017). The application of principal component analysis to drug discovery and biomedical data. Drug Discov. Today 22, 1069–1076. doi: 10.1016/j.drudis.2017.01.005, PMID: 28111329

[ref20] Giuliani A. Filippi S. Bertolaso M. (2014). Why network approach can promote a new way of thinking in biology. Front. Genet. 5:83. doi: 10.3389/fgene.2014.00083, PMID: 24782892 PMC3986556

[ref21] Gutland C. (2018). Denk-Erfahrung. Eine phänomenologisch orientierte Untersuchung der Erfahrbarkeit des Denkens und der Gedanken. Freiburg/München: Alber.

[ref22] Gutland C. (2021). Psychological consciousness of non-psychological contents. Eur. Psychol. 26, 73–84. doi: 10.1027/1016-9040/a000426

[ref23] Gutland C. Wendt A. N. (2023). The struggle to distinguish transcendental phenomenology and psychology. J. für Psychol. 31:1. doi: 10.30820/0942-2285-2023-1-103

[ref24] Heckman C. A. (1990). Geometrical constraints on the shape of cultured cells. Cytometry 11, 771–783. doi: 10.1002/cyto.990110703 2272242

[ref25] Hegel G. W. F. (1977). Phenomenology of Spirit. eds. Findlay J. N. Miller A. V. (Oxford: Oxford University Press).

[ref26] Hegel G. W. F. (1986a). Enzyklopädie der philosophischen Wissenschaften im Grundrisse 1830. Erster Teil. Die Wissenschaft der Logik. eds. Moldenhauer E. Michel K. M.. 10th ed (Frankfurt am Main: Suhrkamp).

[ref27] Hegel G. W. F. (1986b). Enzyklopädie der philosophischen Wissenschaften im Grundrisse 1830. Zweiter Teil. Die Naturphilosophie. eds. Moldenhauer E. Michel K. M.. 8th ed (Frankfurt am Main: Suhrkamp).

[ref28] Hegel G. W. F. (1986c). Phänomenologie des Geistes. eds. Moldenhauer E. Michels K. M.. 13th ed (Frankfurt am Main: Suhrkamp).

[ref29] Hegel G. W. F. (1986d). Enzyklopädie der philosophischen Wissenschaften im Grundrisse 1830. Zweiter Teil. Die Naturphilosophie. eds. Moldenhauer E. Michel K. M.. 9th ed (Frankfurt am Main: Suhrkamp).

[ref30] Hegel G. W. F. (1991). The Encyclopaedia Logic: Part I of the Encyclopaedia of the Philosophical Sciences With the Zusätze. eds. Geraets T. F. Suchting W. A. Harris H. S. (Indianapolis: Hackett).

[ref31] Hegel G. W. F. (2010). The Science of Logic (ed.) Giovanni G. di. Cambridge: Cambridge University Press.

[ref32] Heidegger M. (1993). Phänomenologie der Anschauung und des Ausdrucks. Theorie der Philosophischen Begriffsbildung. ed. Strube C., vol. 59 (Frankfurt am Main: Klostermann).

[ref33] Hume D. (2007a). A Treatise of Human Nature. A Critical Edition. eds. Norton D. F. Norton M. J., vol. 1 (England: Clarendon Press).

[ref34] Hume D. (2007b). An Enquiry Concerning Human Understanding. ed. Millican P. (Oxford: Oxford University Press).

[ref35] Husserl E. (1939). Erfahrung und Urteil. Untersuchungen zur Genealogie der Logik. ed. Landgrebe L. (Prague: Academia).

[ref38] Husserl E. (1938). Phänomenologische Psychologie. Vorlesungen Sommersemester 1925. ed. Biemel W.. 2nd ed (Dordrecht: Springer).

[ref36] Husserl E. (1960). Cartesian Meditations. An Introduction to Phenomenology. ed. Cairns D. (The Hague: Springer).

[ref37] Husserl E. (1962). Die Krisis der europäischen Wissenschaften und die transzendentale Phänomenologie. ed. Biemel W. (The Hague: Martinus Nijhoff).

[ref39] Husserl E. (1970a). Philosophie der Arithmetik. ed. Eley L. (The Hague: Martinus Nijhoff).

[ref40] Husserl E. (1970b). The Crisis of European Science and Transcendental Phenomenology. An Introduction to Phenomenological Philosophy. ed. Carr D. (Evanston: Northwestern University Press).

[ref41] Husserl E. (1973a). Cartesianische Meditationen und Pariser Vorträge. ed. Strasser S.. 2nd ed (The Hague: Martinus Nijhoff).

[ref42] Husserl E. (1973b). Experience and Judgment. Investigations in a Genealogy of Logic. ed. Landgrebe L. (London: Routledge).

[ref43] Husserl E. (1975). Logische Untersuchungen. Erster Band. Prolegomena zur reinen Logik. ed. Holenstein E. (The Hague: Martinus Nijhoff).

[ref44] Husserl E. (1976). Ideen zu einer reinen Phänomenologie und phänomenologischen Philosophie. Erstes Buch. Allgemeine Einführung in die reine Phänomenologie. ed. Schuhmann K. (The Hague: Martinus Nijhoff).

[ref45] Husserl E. (1983). Ideas pertaining to a Pure Phenomenology and to a Phenomenological Philosophy. First Book. General Introduction to a Pure Phenomenology. ed. Kersten F. (The Hague: Martinus Nijhoff).

[ref46] Husserl E. (1984a). Logische Untersuchungen. Zweiter Band. I. Teil. Untersuchungen zur Phänomenologie und Theorie der Erkenntnis. ed. Panzer U. (The Hague: Martinus Nijhoff).

[ref47] Husserl E. (1984b). Logische Untersuchungen. Zweiter Band. II. Teil. Untersuchungen zur Phänomenologie und Theorie der Erkenntnis. ed. Panzer U. (The Hague: Martinus Nijhoff).

[ref48] Husserl E. (2001a). Logical Investigations. eds. Moran D. Findlay J. N., vol. I (London: Routledge).

[ref49] Husserl E. (2001b). Logical Investigations. eds. Moran D. Findlay J. N., vol. II (London: Routledge).

[ref50] Husserl E. (2003). Philosophy of Arithmetic: Psychological and Logical Investigations With Supplementary Texts From 1887–1901. ed. Willard D. (Dordrecht: Springer).

[ref51] Husserl E. (2012). Zur Lehre vom Wesen und zur Methode der eidetischen Variation: Texte aus dem Nachlass (1891–1935). ed. Fonfara D. (Dordrecht: Springer).

[ref52] Jackson F. (1986). What Mary didn’t know. J. Philos. 83, 291–295. doi: 10.2307/2026143

[ref53] Jansen J. (2017). “Eidetik” in Husserl-Handbuch: Leben—Werk—Wirkung. eds. Luft S. Wehrle M. (Stuttgart: J.B. Metzler), 142–148.

[ref54] Kant I. (1998). Kritik der reinen Vernunft. ed. Timmermann J. (Hamburg: Meiner).

[ref55] Kant I. (1999). Critique of Pure Reason. eds. Guyer P. Wood A. W. (Cambridge: Cambridge University Press).

[ref56] Kant I. (2004). Kant: Metaphysical Foundations of Natural Science. ed. Friedman M. (Cambridge: Cambridge University Press).

[ref57] Laughlin R. B. Pines D. (2000). The theory of everything. Proc. Natl. Acad. Sci. U.S.A. 97, 28–31. doi: 10.1073/pnas.97.1.28, PMID: 10618365 PMC26610

[ref58] Laughlin R. B. Pines D. Schmalian J. Stojković B. P. Wolynes P. (2000). The middle way. Proc. Natl. Acad. Sci. U.S.A. 97, 32–37. doi: 10.1073/pnas.97.1.32, PMID: 10618366 PMC26611

[ref59] Lazebnik Y. (2015). Are scientists a workforce? – or, how Dr. Frankenstein made biomedical research sick. EMBO Reports. 16, 1592–1600. doi: 10.15252/embr.201541266 26566662 PMC4693515

[ref60] Locke J. (1997). An Essay Concerning Human Understanding. ed. Woolhouse R. (London: Penguin).

[ref61] Lohmar D. (2005). Die phänomenologische Methode der Wesensschau und ihre Präzisierung als eidetische Variation. Phänomenol. Forsch. 2005, 65–92. doi: 10.28937/1000107912

[ref62] Lohmar D. (2017). “Genetische Phänomenologie” in Husserl-Handbuch: Leben—Werk—Wirkung. eds. Luft S. Wehrle M. (Stuttgart: J.B. Metzler), 149–157.

[ref63] McGee R. J. Warms R. L. (2008). “Postmodernism and its critics” in Anthropological Theory. eds. McGee R. J. Warms R. L. (New York: McGraw Hill), 532–537.

[ref64] Merleau-Ponty M. (2002). Phenomenology of Perception. ed. Smith C. (London: Routledge).

[ref65] Moriarty M. (2008). “Introduction” in Meditations on First Philosophy. With Selections From the Objections and Replies (New York: Oxford University Press), xiii–xxv.

[ref66] Müller J. (1844). Handbuch der Physiologie des Menschen für Vorlesungen, vol. 1. 4th Edn (Koblenz: Hölscher).

[ref67] Newton I. (1952). Opticks. ed. Bell G. (New York: Dover).

[ref68] Plessner H. (1975). Die Stufen des Organischen und der Mensch. Einleitung in die philosophische Anthropologie. 3rd. Edn. (Berlin/New York: de Gruyter).

[ref69] Popper K. (2002). The Logic of Scientific Discovery (2nd Edn.). (London: Routledge).

[ref70] Quine W. V. O. (1951). Main trends in recent philosophy: two dogmas of empiricism. Philos. Rev. 60, 20–43. doi: 10.2307/2181906

[ref71] Richter K. Rost J.-M. (2002). Komplexe Systeme (Frankfurt am Main: Fischer).

[ref72] Scheler M. (1976). “Der Mensch im Zeitalter des Ausgleichs” in Späte Schriften. ed. Frings M. S., vol. 9 (Bern: Francke), 145–170.

[ref73] Scheler M. (2018). Die Stellung des Menschen im Kosmos. ed. Henckmann W.. 1st ed (Hamburg: Meiner).

[ref74] Shear J. (ed.) (1999). Explaining Consciousness: The Hard Problem. Cambridge: Bradford.

[ref75] Shields C. (2011). “On behalf of cognitive qualia” in Cognitive Phenomenology. eds. Bayne T. Montague M. (Oxford: Oxford University Press), 215–235.

[ref76] Sowa R. (2010). Husserls Idee einer nicht-empirischen Wissenschaft von der Lebenswelt. Husserl Stud. 26, 49–66. doi: 10.1007/s10743-010-9067-5

[ref77] Stähler T. (2003). Die Unruhe des Anfangs. Hegel und Husserl über den Weg in die Phänomenologie. (Berlin: Springer).

[ref78] Staiti A. (2017). “Wissenschaftstheorie” in Husserl-Handbuch: Leben—Werk—Wirkung. eds. Luft S. Wehrle M. (Stuttgart: J.B. Metzler), 173–178.

[ref79] Ströker E. (1979). “Geschichte und Lebenswelt als Sinnesfundament der Wissenschaften in Husserls Spätwerk” in Lebenswelt und Wissenschaft in der Philosophie Edmund Husserls (Frankfurt am Main: Klostermann), 107–123.

[ref80] Tye M. (2021). “Qualia” in The Stanford Encyclopedia of Philosophy (fall 2021). ed. Zalta E. N. (Stanford: Metaphysics Research Lab).

[ref81] Urry L. A. Cain M. L. Wasserman S. A. Minorsky P. V. Reece J. B. (2021). Campbell Biology (12th). Hoboken: Pearson.

[ref82] Waldenfels B. (2006). Grundmotive einer Phänomenologie des Fremden. Frankfurt am Main: Suhrkamp.

[ref83] Weaver W. (2004). Science and complexity. Emerg. Complex Organ. 6, 65–74.

[ref84] Wittgenstein L. (1974). Bemerkungen über die Grundlagen der Mathematik. Revidierte und erweiterte Ausgabe. (eds.) Wright G. H. von Anscombe G. E. M. Rhees R. Frankfurt am Main: Suhrkamp.

[ref85] Ziegler R. Weger U. (2023). Thinking action as a performative and participative mental awareness. Front. Psychol. 14:901678. doi: 10.3389/fpsyg.2023.901678, PMID: 37205059 PMC10185780

